# Career motivation and burnout among medical students in Hungary - could altruism be a protection factor?

**DOI:** 10.1186/s12909-016-0690-5

**Published:** 2016-07-18

**Authors:** Zsuzsa Győrffy, Emma Birkás, Imola Sándor

**Affiliations:** Institute of Behavioural Sciences, Semmelweis University, Nagyvárad Square 4, Budapest, H-1089 Hungary

## Abstract

**Background:**

Burnout is a major issue among medical students. Its general characteristics are loss of interest in study and lack of motivation. A study of the phenomenon must extend beyond the university environment and personality factors to consider whether career choice has a role in the occurrence of burnout.

**Methods:**

Quantitative, national survey (*n* = 733) among medical students, using a 12-item career motivation list compiled from published research results and a pilot study. We measured burnout by the validated Hungarian version of MBI-SS.

**Results:**

The most significant career choice factor was altruistic motivation, followed by extrinsic motivations: gaining a degree, finding a job, accessing career opportunities. Lack of altruism was found to be a major risk factor, in addition to the traditional risk factors, for cynicism and reduced academic efficacy. Our study confirmed the influence of gender differences on both career choice motivations and burnout.

**Conclusion:**

The structure of career motivation is a major issue in the transformation of the medical profession. Since altruism is a prominent motivation for many women studying medicine, their entry into the profession in increasing numbers may reinforce its traditional character and act against the present trend of deprofessionalization.

## Background

The first published account of student burnout was by Kafry and Pines [1]: they evaluated as “burnout” the condition of reduced interest in studies, lack of motivation and fatigue. The next stage in student burnout research was the determination of predisposing factors. These were grouped into external (university workload and environment) and internal (personality) factors; their mutual interactions were also assumed to be important in the formation of burnout. The external causes found to be most significant are the quantity of material to be learned, pressure of time, examination stress and financial uncertainty. Being confronted with death and suffering and other emotional burdens is also involved in student burnout [2–5]. Another factor is fear of dropping out or failing university [6, 7]. Studies have found a clear link to the personality (internal) factors self-confidence, self-effectiveness, control, anxiety and neuroticism [8–11]. Related to this finding is the application of the battered child syndrome to describe the difficulties experienced by medical students. This refers to the feelings of shame and frequent criticism experienced in the course, low sense of control, and defensive reactions to feelings of helplessness and dependence [12].

The prevalence of student burnout does not emerge consistently across different studies. Early reports put it between 10 and 45 % [12], but more recent research has found about half of students to be affected [13]. These studies have found burnout to be more frequent among medical students than their peers in other university courses [14].

The consequences of student burnout are as complex as the background factors mentioned above. Many mental distress factors (anxiety, sleep disorders, depression and suicidal ideation) appear among students suffering from burnout [12, 15–18]. Several studies share the finding that burnout has a close relationship with smoking, alcohol consumption and unhealthy lifestyle (unhealthy eating, lack of exercise) [19–21].

There is thus a very broad spectrum of background factors in student burnout. Very few studies, however, have focused on the relationship between student burnout and career choice and motivation. Pagnin and De Queiroz [22] found that students whose career choice motivations included the illness or death of a relative scored much higher in the emotional exhaustion component of burnout than students with other motivations.

Knowledge of career choice factors and motivations could shed light on the deprofessionalization of medicine which has been going on for about fifty years. One of the most characteristic themes in deprofessionalization is the decline in the authority of doctors and of trust in them. Doctors nowadays are not so much charismatic healers as “service professionals”, taking their places alongside paramedics and complementary practitioners. The doctor-patient relationship is a prominent element in deprofessionalization [23, 24]. Changes in medical practice certainly have an influence on the perceptions and motivations of young people choosing a career. Altruism characterized by a desire to help others, is mentioned as the overwhelmingly dominant motivation factor in the literature on the subject from the 1950s to the 1970s. Altruism is seen as enabling the professional self to develop [25].

The earliest treatment of the subject [26] saw the most important motivations for choosing a medical career to be altruism and family (i.e. doctor parents). This view was confirmed by many further studies [27]. A longitudinal study by Hyppölä et al. [28] detected the high prestige of the profession and the wide range of occupational opportunities as further important factors. McManus listed five primary factors affecting choice of career: aptitude for science subjects, career opportunities, the influence of friends and relations, the desire to help people and the reason, “as long as I’ve known my own mind, I’ve wanted to be a doctor” [29]. A recent study by Shankar et al. has pointed up the significant of family influences on career choice [30].

Our study focuses on the little-explored link between career choice motivations and student burnout. Starting out from relevant findings in the literature [31, 32], we proposed a linear relationship between students’ altruistic commitment and burnout: altruistic motivation generates greater involvement, commitment and emotional burden, which can induce a higher level of emotional exhaustion, cynicism and reduction of academic performance.

## Methods

We used an anonymous self-completing questionnaire to survey students of general medicine in the four Hungarian medical universities (Budapest, Debrecen, Pécs and Szeged) in academic year 2012/2013. The main sampling criterion was the inclusion of students in both preclinical and clinical training in similar proportions. Students in what have been identified as the “watershed” 1st, 3rd and 5th years of study [33] were included in relatively high proportions. Our survey was conducted among Hungarian medical students and our sampling frame was 1300. The response rate was 733 (56 %). Three hundred participants were chosen from 3 universities respectively. In Budapest, we have asked 400 students. (Semmelweis University, the biggest medical university in Hungary.) The response rates were respectively: Budapest 345 (86.25 %), Pécs:163 (54 %), Debrecen: 33 (11 %), Szeged:192 (64 %).

We didn’t have detailed statistical data on the sociodemographic composition of the students that would have enabled subsequent weighting for representativeness, and so the generalization of our results to the Hungarian medical student population as a whole is limited. We conducted the study with permission from the Semmelweis University Ethics Committee. (Ethics permit number: 2013/59).

The questionnaire concerned physical and mental health, health behaviour, stress load and coping strategies among Hungarian medical students, and their career motivations. The design of the questionnaire largely drew on our previous research among doctors [34, 35].

The questions were grouped as follows:

1. Demographic details (gender, age, year of study, university).
2. Health details (list of psychosomatic symptoms, self-estimate of health).
3. Psychological factors (depression, sleep disorders, suicidal behaviour, burnout, empathic attitude, parental bonding).
4. Health behaviour (smoking, alcohol consumption, exercise).
5. Career background factors (doctor parents, time of choice of career, career choice motivations).
6. Stressors during the years at medical university (stress factors, examination stress, overload, coping, dissection-related stressors).

### Measuring instruments

We carried out a pilot study among 175 medical students to determine career choice motivations. They were asked to write the story of their career choice in terms of given criteria, in 1–2 pages. The texts were analysed using qualitative (content analysis) and quantitative (SPSS.PC 20.1) methods. All career choice motivating factors were gathered from the texts. The two reduced lists thus generated (grouped in 12 categories) were compared, the divergent indications were discussed and a final list was drawn up based on consensus interpretation. A twelve-item category system [36] was produced out of the aggregated motivation list. The twelve career motivation categories were: gaining a degree, attractiveness of student life, altruism, effect of doctor parents, prestige of the profession, illness of a close relative or friend, interest in admission (science) subjects, high earnings, ease of finding a job, work abroad, positive health example, and negative health example.

To measure burnout, we used the validated Hungarian version of the Maslach Burnout Inventory (MBI SS) [37–39]. The self-completing questionnaire consists of 15 questions arranged around three factors: emotional exhaustion (5 items), cynicism (4 items) and reduced academic efficacy (6 items). The items concern how burdensome the students feel university studies to be, and how often and how intensely they have experienced certain conditions over the last three months [38]. Respondents marked their answers on a 0–6 Likert scale, and the scores were analysed by dividing them into the bands recommended by international standards and Hungarian validation [38]. Cronbach-alpha was 0.804 for emotional exhaustion, 0.852 for cynicism and 0.815 for reduced academic efficacy.

### Statistical methods

From descriptive analyses, we calculated frequency, average and standard deviation. We also noted the percentage differences between variables. Depending on the variable type, we used the independent sample *t*-test and chi-square test. Multivariate analysis involved binary logistic regression to examine the interdependence of student burnout and career motivation factors. The dependent variable was a two-category version of burnout (low/moderate and severe), and the control variables were the primary student burnout-- factors defined in the literature: gender, stage of study (preclinical/clinical) and satisfaction with study outcomes (on a 1–5 Likert scale where 1 was “very dissatisfied” and 5 “completely satisfied”) and time of career choice (before or after the age of 14). The Likert-scale variables were grouped as dichotomous variables. Another control variable was overload (0 not at all, 1 highly), and stressors connected to quantity of learning and examinations. (What stressors are there in your life? Please rate them between 1 and 5, where 1 means that the factor is not a stressor, and 5 that it is a serious stressor. The final step in the model was to introduce career motivation factors showing a connection with the univariate analysis. (Job opportunities, foreign work opportunities, financial, altruism). We used the SPSS 20.1 program for statistical analysis.

## Results

### Demographic data

There were 733 respondents in the survey. The sample had a gender distribution of 33.2 % (243) men to 66.8 % (488) women, and was divided roughly half-and-half between preclinical (1 and 2) and clinical (3 and 4) years (48 % vs. 52 %) (Table 1).

**Table 1:** Distribution of the sample by gender and year of study (*n* = 721)

Year	Total		Women		Men	
	%	*n*	%	*n*	%	*n*
I.	21.8	157	60.5	95	39.5	62
II.	26.2	189	69.8	132	30.2	57
III.	25.4	183	70.5	129	29.5	54
IV.	7.1	51	60.8	31	39.2	20
V.	1.9	14	64.3	9	35.7	5
VI.	17.6	127	67.7	86	32.3	41
		721		482		239

The average age of students in the sample was 22.4 years (SD = 2.14). The distribution among universities was 47.1 % (345) Budapest, 4.5 % (33) Debrecen, 26.2 % (192) Pécs and 22.2 % (163) Szeged.

### Vocation, career motivation

From the twelve-item list of motivations derived from the pilot study, we formulated statements that expressed respondents’ career choice motivations: “I would definitely like to get a degree. I find student life attractive. I would like to help people. My parents are doctors. I was attracted by the prestige of the profession. Because of the illness of a close relative. The admission subjects were an influence. With a medical degree it is easy to get a job/you can make a good living/you can get work abroad. Because of positive or negative health experiences.”

The results show that altruism, “the wish to help people”, is what most of all prompts students to study medicine (564 respondents, 77 %). The wish to gain a degree came second (392, 53.6 %), the “ease of getting a job”, third (329, 45 %) the high prestige of the medical profession, fourth (292, 39.9 %), and the option of working abroad, fifth (238, 32.9 %). Interestingly, the influence of doctor parents had the smallest part in respondents’ motivation (85, 11.6 %). Illness of a closer relative or friend was also seldom mentioned as a career motivation factor (99, 13.5 %).

Several of the career motivation related questions revealed substantial differences between men and women. Women gave altruism as the reason for their career choice significantly more often than men (*p* < 0.015), and men were significantly more likely to indicate the effect of doctor parents (*p* < 0.020), professional prestige (*p* < 0.002), potential future earnings (*p* = 0.002), and good job opportunities (*p* = 0.040) (Table 2).

**Table 2:** Gender differences in the distribution of reasons given for career choice

Career choice reasons	Total		Women		Men		*χ* ^2^ test
	%	*n*	%	*n*	%	*n*	*p*
altruism	76.9	562	79.5	388	71.6	174	0.02
extrinsic motivations							
gaining a degree	53.5	391	54.1	264	52.3	127	ns.
earnings potential	18.6	136	15.4	75	25.1	61	0.00
work abroad	32.6	238	32.0	156	33.7	82	ns.
ease of getting a job	45.0	329	42.2	206	50.6	123	0.04
professional prestige	39.9	292	35.9	175	48.1	117	0.00
other							
doctor parents	11.6	85	9.6	47	15.6	38	0.02
attractiveness of student life	24.1	176	23.6	115	25.1	61	ns.
illness or death of close relative	13.5	99	13.5	66	13.6	33	ns.
admission subjects	27.1	198	26.2	128	28.8	70	ns.
positive medical example	11.8	86	11.5	56	12.3	30	ns.
negative medical example	10.0	73	10.9	53	8.2	20	ns.

*ns.* not significant

Since students could indicate multiple, unranked career choice motivations, we grouped motivations into three groups: an intrinsic factor (altruism), extrinsic factors (gaining a degree, finding a job, foreign work opportunities, prestige and potential earnings) and others (interest in admission (science) subjects, attractiveness of student life, effect of doctor parents, illness of a close relative or friend, positive health example, and negative health example). This is shown on Table 3. We found that 115 students indicated altruism and no others, i.e. no extrinsic motivations. 142 indicated only extrinsic motivation factors, and thus not altruism. The majority of students (447) indicated both altruism and extrinsic motivations. Thus consideration of multiple factors dominates in students’ motivational structure, and altruism occurs most frequently among these multiple factors.

**Table 3:** Relation between career choice reasons and stage of study

Carrier choice reasons	Total		Preclinical Group		Clinical Group		*χ* ^2^ test
	%	*n*	%	*n*	%	*n*	*p*
altruism (intrinsic motivations)	77.8	561	81.6	283	74.3	278	ns.
extrinsic motivations							
gaining a degree	53.5	386	54.2	188	52.9	198	ns.
earnings potential	18.4	133	22.5	78	14.7	55	0.01
work abroad	32.6	265	36.3	126	29.1	109	0.04
ease of getting a job	44.8	323	46.1	160	43.6	163	ns.
professional prestige	39.5	285	39.2	136	39.8	149	ns.
other							
doctoral parents	11.7	84	12.4	43	11.0	41	ns.
attractiveness of student life	24.5	177	29.1	101	20.3	76	0.01
illness or death of close relative	13.5	97	15.0	52	12.0	45	ns.
admission subjects	27.2	196	22.8	79	31.3	117	0.01
positive medical example	85	11.8	13.3	46	10.4	39	ns.
negative medical example	10.0	72	10.4	36	9.6	36	ns.

*ns.* not significant

We examined the connection between career choice factors and stage of study (preclinical/clinical). Interest in admission subjects (science) was significantly higher among students in clinical years (*p* < 0.012) and opportunity to work abroad was significantly higher among those in the preclinical phase (*p* < 0.004) (Table 3).

### Student burnout

Table 4 shows the frequencies of burnout scores in the low and medium/severe burnout ranges.

**Table 4:** Burnout indicators of the sample

Burnout dimensions	Low		Medium and high	
	%	*n*	%	*n*
Emotional exhaustion	61.4	438	38.6	275
Cynicism	65.5	472	34.0	249
Reduced professional efficacy	76.0	544	24.0	172

Women were found to be affected by emotional exhaustion to a significantly greater extent than men (32.1 % vs. 41.8 %, *p* < 0.012). There were no gender differences in the dimensions of cynicism and reduced academic efficacy (not shown in a table).

Stage of training (preclinical/clinical) had no influence on emotional exhaustion or reduced academic efficacy, but significantly more students in clinical training experienced moderate and severe cynicism (38.5 % vs. 61.5 % *p* < 0.001) (not shown in a table).

### Connections between career choice factors and burnout indicated by univariate analysis

Moderate and severe emotional exhaustion displayed a statistically non-significant association with earnings potential and opportunities to work abroad. Students who did not indicate altruism were more likely to be in the moderate and severe ranges of cynicism (47.7 vs 30.9 *p* < 0.001). Cynicism was also more severe among those who indicated ease of employment (31.8 % vs. 38.8 % *p* < 0.028) and foreign work opportunities (31.8 % vs 40.1 *p* < 0.030). Students who did not indicate altruism were also more likely to have moderate or severe reduction of academic efficacy (33.5 % vs. 21.2 % *p* < 0.002). Another statistically non-significant tendency is greater reduction of academic efficacy among those indicating work opportunities abroad (22.1 % vs. 27.8 % *p* < 0.095).

### Connections between career choice factors and burnout indicated by multivariate analysis

Factors known to influence burnout [2] include examination stress, overload, satisfaction with study outcomes and difficulties in concentrating on studies. The next stage was therefore a binary logistic regression analysis to determine how burnout is related to career motivations and other factors. We found that emotional exhaustion was not related to any of the career motivations, and related most strongly to examination stress, overload and difficulties in concentrating on studies (not shown in a table).

In the cynicism dimension of student behaviour, burnout, the absence of the altruistic career motivation remained a significantly determining factor (Table 5). Medium and high values of this variable occur in higher proportions in later clinical years and among those indicating overload. The variance explained by the model (Nagelkerke R2) is 15.6 %.

**Table 5:** Variables explaining the cynicism/depersonalization factor of burnout in the binary logistic regression model

Career choice motivation	Cynicism/depersonalization		
	Exp (B)	95 % CI	*p*
Gender	0.89	0.61 to 1.28	0.52
Year of study (preclinical/clinical)	1.96	1.35 to 2.77	0.00
Time of carrier choice(after/before 14)	0.82	0.57 to 1.59	0.31
Satisfaction with studies	1.65	0.80 to 1.65	0.44
Examination stress	0.71	0.44 to 1.14	0.16
Concentration stress	0.74	0.52 to 1.08	0.12
Work overload	2.44	1.64 to 3.70	0.00
Getting a job	1.15	0.78 to 1.69	0.49
Work abroad	1.09	0.72 to 1.64	0.69
Money	1.07	0.66 to 1.72	0.80
Altruism	1.92	1.30 to 2.86	0.00

For the reduced academic efficacy factor of burnout, the probability of medium and severe performance reduction is increased by lack of altruistic career motivations and significantly reduced by satisfaction with studies (Table 6). The variance explained by the model (Nagelkerke R2) is 14 %.

**Table 6:** Variables explaining the reduced academic efficacy factor of burnout in the binary logistic regression model

Career choice motivation	Reduced Academic Efficacy		
	Exp (B)	95 % CI	*p*
Gender	0.77	0.51 to 1.17	0.22
Year of study (preclinical/clinical)	1.03	0.69 to 1.54	0.89
Time of carrier choice (after/before 14)	1.31	0.87 to 1.96	0.20
Satisfaction with studies	3.41	2.27 to 5.12	0.00
Examination stress	0.61	0.35 to 1.05	0.07
Concentration stress	0.80	0.52 to 1.23	0.31
Work overload	1.00	0.64 to 1.56	1.00
Getting a job	0.85	0.55 to 1.33	0.47
Work abroad	1.26	0.79 to 1.99	0.34
Money	0.86	0.50 to 1.47	0.58
Altruism	1.69	1.09 to 2.63	0.02

## Discussion

We asked 733 medical students about their career choice motivations and explored the occurrence of burnout among them. The results show that the most common career choice factor is altruistic motivation, followed four extrinsic motivations: gaining a degree, getting a job, career opportunities and secure earnings. We found differences in the motivational structure of men and women: female students were more likely to indicate the “I would like to help people” motivation. Students in clinical years were more likely to mention admission subjects, and those in preclinical years foreign work opportunities. The investigation of components of burnout found relatively high levels of emotional exhaustion among women and of cynicism among students in the clinical phase of the course. Both uni- and multivariate analysis confirmed the relationship between career choice motivations and the medium/severe range of burnout: lack of altruism may be added to the known major risk factors for cynicism and reduced academic efficacy.

The three burnout variables for which high proportions of the overall sample were found to be in the moderate/severe range were emotional exhaustion (almost 40 %), cynicism (almost one third), and reduced academic efficacy (over 20 %). These results confirm that Hungarian medical students are at a high risk of burnout [16, 40, 41]. Similar to international studies, they find that burnout increases with stage of study: severity according to all burnout indicators is greater in the clinical than the preclinical periods [40, 42]. Different studies have made strikingly contradictory findings in respect of gender: in some, men display more severe cynicism [43, 44] and women more severe emotional exhaustion; others have found no gender differences in burnout [40, 42]. However, methodological problems such as differences in measuring instruments and cut-off points cause difficulties for comparisons of student burnout. Another obstacle to comparison arises from patterns of medical training. In much of Europe (including Hungary), many students enter medical training at the ages of 18 or 19, whereas in the USA and Australia, students usually enter a medical course after receiving a BA [16, 42]. Differences in stress sensitivity, burnout indicators and coping may be due to age and “experiential differences”.

The next stage of analysis concerned career choice motivations. Altruism is still a very important factor both by individual comparison and after grouping by extrinsic/intrinsic criteria, but others are also important. About the same number as indicated a wish to help also included factors that fit the concept of “controllable lifestyle” [45, 46] – money, finding a job, and career opportunities – were indicated by respondents with about the same frequency as the “wish to help”. This implies that altruism, despite its importance, is not the only consideration for students; plans for career and status also feature in high proportions. This is in line with international trends [27]. We must also consider the question of how, in 21st century medicine, altruism can constitute a motivation. Technological developments have created new forms of doctor-patient relationship: in many branches of the profession, the possibilities for helping via a less personal doctor-patient relationship are much more numerous than they were 50 or 60 years ago. A finding in Hungarian studies that nearly half of first year students do not want to enter clinical practice highlights another important development [47]. The changing medical profession now offers many opportunities in such areas as research and marketing, requiring a fundamental change in the picture of the helping, healing doctor standing beside the sickbed.

There are also significant gender differences in motivations. Our finding that altruism is of greater moment in the motivational structure of women than of men is consistent with many international studies ([48, 49]. In a much-cited study by Johansson and Hamberg [48] Swedish medical students were asked to write essays where they imagined themselves as practising doctors. The helping, self-sacrificing attitude (Florence Nightingale) tended to show up more in women’s imagination, and the image of the effective, practical, resolute doctor (Superman or Doctor House) in men’s. Gender aspects of career choice are taking on increasing significance in the light of the feminization of the profession. The number of female doctors has increased dramatically in recent decades; in the United States, the proportion of women among medical students has gone up from about 10 % fifty years ago to more than 50 % today [50]. The trend also shows up in the gender distribution of applications for medical courses: there was a clear male majority in the 1950s and 1960s, numbers were approximately equal in the 1970s and 1970s, and by the 1990s and the first decade of the 21st century, applicants were predominantly female [51–53]. Forecasts made in the 1990s put the likely proportion of women doctors by 2010 as one third, but nearly half of those choosing and practising a medical career are now women [54, 55]. In medical universities throughout the world, women now account for more than 60 % of students [56, 57]. Exploration of this feminization and the background of career choices has shown up an interesting feature of change in the profession. The trend has been accompanied by the phenomenon of deprofessionalization, while women’s early commitment and altruistic-based choice of profession reinforces the traditional nature of the profession, and so acts in favour of reprofessionalization.

A central question of our study was whether and how career choice motivations relate to different dimensions of burnout. Multivariate analysis showed that overload, examination stress and difficulties of concentrating on study are dominant in student burnout, but altruism is also an important factor. The mechanisms by which altruistic motivations and burnout interact remain an open question. These may be approached via several considerations. Altruism is a strong intrinsic motivation which may protect students when confronted with the difficulties of university life: “if I can see my objective, I can better adapt to the difficult circumstances”. Another possible explanation is that those who indicate altruism as a motivation are in a different stage of “career socialization” than those who do not (the same could be said for men and women). Our other studies have shown that girls commit themselves to a medical vocation at an earlier stage than boys [58]. Earlier choice could indicate a more conscious commitment and be an advantage in the study-related burnout indicators. This is confirmed another finding of our 2012 research that a higher proportion of boys than girls have doctors as parents: meeting and idealizing parents’ expectations could also be an explanatory factor in the higher rate of burnout among male students.

A strength of our study is its focus on an aspect of student burnout that has hitherto attracted little attention. It has clearly shown that scepticism regarding the purpose of study (cynical attitude) is incompatible with altruistic motivations. One limitation is its cross-sectional nature, which does not give the chance to study causal issues. Another is that the measuring instrument of student burnout only looks at study-related aspects, whereas student burnout is likely to have other important dimensions. An additional limitation of our study is the generalization of our results to the Hungarian medical student population as a whole is limited. Debrecen provided fewer evaluable responses than any other of the four Hungarian universities with medical faculties, and this indicates a need for further research. Our study opens the way to many new areas: beyond the exploration of career choice motivations, a major question for future research is the gender aspect of career choice. The issues connected with the ongoing feminization of the profession must be taken heed of in future medical training.

## Conclusion

The structure of career motivation is a major issue in the transformation of the medical profession. Since altruism is a prominent motivation for many women studying medicine, their entry into the profession in increasing numbers may reinforce its traditional character and act against the present trend of deprofessionalization.

## Abbreviation

MBI SS, Maslach Burnout inventory student version
